# Trump tariffs and the U.S. defense industry

**DOI:** 10.1371/journal.pone.0313204

**Published:** 2025-01-24

**Authors:** Jeroen Klomp

**Affiliations:** 1 Netherlands Defense Academy, Breda, The Netherlands; 2 Wageningen University and Research, Wageningen, The Netherlands; Middle Tennessee State University Jennings A Jones College of Business, UNITED STATES OF AMERICA

## Abstract

In March 2018, U.S. President Trump announced that the U.S. would start imposing tariffs on steel and aluminum imports from most exporting countries around the world. This study explores the impact of introducing these tariffs on the equity return of U.S. defense companies. As the defense industry stands among the largest metal consumers in the U.S., it is expected that these import restrictions have deteriorated the business performance of the U.S. defense industry. For this study, a novel trade uncertainty indicator has been constructed that is based on the key events related to the invocation of Section 232 of the Trade Expansion Act. This section empowers the President to impose trade restrictions when the quantity of imports threatens to impair national security. My empirical analysis reveals that investors perceived the introduction of the steel and aluminum tariffs as detrimental to U.S. defense companies. The negative abnormal stock returns in the days around several key tariff-related events evidence this. Already in the period before the Department of Commerce released the findings of its investigation, investors were speculating on the possible introduction of trade barriers. However, the height of the imposed tariff exceeded their expectations since the negative sentiment was further reinforced after the official announcement of the tariff by President Trump.

## 1. Introduction

The U.S. defense industry plays a pivotal role in ensuring national security and bolstering the economy. Economically, it generates sales revenue of over $700 billion annually, thereby contributing to about ten percent of the total exports [1]. The defense industry employs millions of high-skilled U.S. workers and drives significant advancements in science and engineering through substantial research and development investments. These innovations often spill over into the civilian sector, fostering broader economic growth and enhancing the nation’s competitive edge in various industries [2]. Strategically, the defense industry provides the technological advancements and sophisticated weaponry essential for maintaining technological leadership and global military dominance. By equipping the armed forces with cutting-edge tools and systems, the defense sector helps safeguard national interests and protect against a wide array of threats, ranging from traditional military conflicts to cyber warfare and terrorism [3].

Steel and aluminum are critical materials in the production of major conventional weapons. The U.S. defense industry ranks among the largest consumers of both metals, and having unfettered access to them is pivotal for producing U.S. military systems [4]. This is especially true for certain very strong alloys and specially manufactured grades of steel and aluminum. The outstanding capabilities of many U.S. weapons systems depend upon these exceptionally strong and highly engineered materials, including nuclear-powered aircraft carriers, submarines, tanks, armoured vehicles, and advanced fighter jets. Therefore, U.S.-made steel and specialty metals are recognized as vital components of U.S. military strength and capabilities [3].

While the United States has numerous steel-producing allies, the reliance on foreign-owned facilities located outside the United States poses significant risks and potential delays for the development of new steel products, particularly in times of emergency [5–8]. This underscores the intertwined nature of political discussions about national security and steel production. In 2018, U.S. President Donald Trump reignited this debate by invoking Section 232 of the Trade Expansion Act. This section empowers the President to impose trade barriers when the quantity of imports poses a threat to national security. The application of this act resulted in a tariff rate of 25 percent on steel imports and 10 percent on aluminum imports from most exporting countries, further underscoring the potential risks of foreign reliance [9].

The justification of the application of Section 232 was based on the assertion of the government that domestic production of steel and aluminum is essential for the production of defense items and that protecting the steel industry is therefore vital for national security interests [10,11]. However, many academic scholars and political analysts contended that domestic politics played a dominant role in this matter. In particular, Section 232 tariffs provide a classic textbook example of how politically powerful and well-organized special interest groups secure government favors by passing on the costs to the general population. The Trump administration chose to protect only one small part of an industry with a much broader reach and impact on the U.S. economy [12–14].

More broadly, the application of Section 232 fits a recent global trend of using trade policies for non-economic objectives. Hoekman et al. [15] demonstrate, based on data taken from the Global Trade Alert, that national security concerns are one of the most important non-economic reasons to impose quantitative trade restrictions in the last decade. This trend has caused a serious reassessment of maintaining liberal trade and investment relations with potential adversaries. Countries started to implement measures to safeguard strategic and national autonomy, including enhancing the resilience of global value chains to geopolitical shocks and diversifying the sourcing of critical goods and services. Unilateral actions by countries seeking to ensure competitive neutrality and preserve autonomy aim to decouple from other countries, especially from China. There is currently a small, but fast-growing body of studies exploring the use of trade policies in the strategic competition among countries (see, e.g., [16–21]). This paper aims to contribute to this literature.

One common way to discern how companies are affected by the introduction of the tariff scheme is by examining the dynamics in the stock market return. The equity value of a company is based on the discounted infinite stream of dividends. Under the semi-strong form of the efficient market hypothesis, equity prices are assumed to reflect all public information and to adjust swiftly to the arrival of new public information that is relevant to the expected future earnings of the firm [22–26]. A growing body of empirical studies has analyzed the introduction of these so-called Trump tariffs on the stock market performance of companies (see, among others, [27–34]). All of these studies arrive at the common conclusion claiming that protectionist policies diminish the market value of U.S. companies by negatively affecting investor sentiment, regardless of whether these firms rely primarily on domestic sales or have a more export-oriented focus.

However, most previous studies explore the general effect of the steel and aluminum tariffs on the stock market performance using a diverse panel of industries or companies. It is, however, questionable whether the economic costs and/or benefits of the tariff are shared equally among the economic sectors driving the U.S. economy, given the different exposure to these tariffs among industries. In turn, this study, focuses specifically on the defense industry, as these companies are typically being omitted in previous studies. Yet, the companies in this sector share some distinct characteristics that set them apart from other large-scale aluminum or steel users. First, the defense-related industry is frequently susceptible to geopolitical risks, including trade disputes and political conflicts, as strategic and political considerations heavily influence the domestic and export demand in this sector [35–38]. In the wake of this risk, investors in the defense sector typically quickly reshuffle their portfolio when the likelihood of geopolitical risks materializing escalates. Second, the defense industry is one of the largest consumers of steel and aluminum, as it is responsible for about ten percent of the total steel and aluminum demand in the United States. For many defense companies, the steel and aluminum price determine their production costs for over a quarter [39]. However, unlike other large steel and aluminum-using industries, such as the construction or automobile industry, the defense industry almost exclusively uses domestically produced steel. This is particularly true for certain very strong alloys and specially manufactured grades of steel and aluminum. Lastly, the U.S. defense industrial base largely differs from other arms-producing countries. The U.S. defense industry possesses substantial market power due to economies of scale and comparative technological advantages. This limits the substitution possibilities of trading partners to rely on alternative arms-exporting countries, as a retaliation strategy, for their arms imports [2].

This paper investigates the impact of introducing the tariff scheme on the expected profitability of the U.S. defense industry and whether this effect is sector-specific. For this purpose, I employ various empirical analyses to test if the main events related to the tariff scheme have resulted in abnormal stock returns for U.S. defense companies. More specifically, I have combined a classic (non)parametric and nonparametric analysis together with a panel regression analysis [40–42]. Additionally, I have constructed a new dataset about the trade policy uncertainty that resulted from invoking Section 232 of the Trade Expansion Act by President Trump. This dataset is based on the number of related news articles published by major international newspapers, thereby identifying the key event dates related to introducing and implementing the tariff scheme. In addition, I used the abnormal stock market return of about sixty publicly listed U.S. defense companies between January 2017 and December 2020.

My analysis reveals that investors perceived the introduction of the steel and aluminum tariffs by President Trump as a negative event for the market value of U.S. defense companies. The drop in the abnormal stock market returns is likely to be caused by a decrease in the expected companies’ future cash flows, higher production costs, and the fear of retaliation actions taken by important trading partners. Even before the Department of Commerce released the findings of its investigation and President Trump had officially announced the tariff, investors were already speculating on the introduction of steel and aluminum trade restrictions. However, the actual tariff rate introduced exceeded expectations since the negative sentiment was aggravated after the official announcement of the tariff by President Trump. It turns out that the tariff-related events reduced the stock price in total by about three percent in the period of my analysis. However, the impact found varies substantially across companies. Companies producing steel-abundant defense items suffer more from the tariffs. But even for these companies, the impact is moderate. One rational explanation is that the U.S. defense industry has a monopoly on the export market, making retaliation actions less successful. Finally, comparing the U.S. defense industry with foreign competing companies or other large steel-consuming companies reaffirms this unique role.

These findings are relevant not only from an academic perspective, but perhaps even more critically, from a policy view. The results of this research indicate a substantial trade-off—protecting the domestic steel industry adversely impacts the U.S. defense industry [43]. This observation challenges President Trump’s rationale for shielding the steel industry, which was ostensibly to achieve political gains while simultaneously ensuring a secure supply of steel and aluminum for the defense sector to bolster national security. The critical question is whether these dual objectives can be harmonized, given that externalities, such as increased domestic steel prices and retaliatory measures from trading partners, may impose costs on the U.S. defense industry, thereby weakening it.

The remainder of the paper is structured as follows. The next section provides the theoretical background underlying my research question, while in section three, I present my data and methodology used. In sections four and five, I respectively report my estimation results of the classic event analysis and a regression model. Finally, I end in section six with my conclusions and discussion.

## 2. Background

The United States is the world’s largest steel importer, as it is responsible for about ten percent of the global steel imports. In particular, the U.S. imports four times more steel than it exports and about a quarter more than Germany—the world’s second-largest importer [39]. The national character of the U.S. steel industry in an environment of intense international competition provides the basis for reflex protectionist actions when imports impinge on domestic steel markets. Consequently, steel trade policies have been a major political issue for decades in the U.S., especially since the steel industry is highly concentrated, well organized, and located in important “must-win” electoral districts. As a result, the steel industry in the United States has been extremely protected from foreign competition for decades (see also [44]). Various arguments have been put forward for protecting the steel industry, including the infant industry argument, countercyclical protection, protection from unfair trade practices, and national security concerns [4]. Particularly, import protection for the steel industry under the umbrella of national defense can be viewed as a form of insurance policy, ensuring a usable steel capacity in case foreign supplies are disrupted. However, several considerations cast doubt on the efficacy of such a policy. From a practical point of view, trade protection may, in fact, directly contradict the goal of national security by enabling an uncompetitive industry to avoid necessary adjustments and compete on quality. Advocates of trade protection argue that steel requirements by the military in times of war will exceed the steelmaking capabilities of the industry in peacetime [8]. However, the empirical support for this latter claim is only weak. In the case of the United States, the evidence suggests that potential defense needs do not require the maintenance of capacity above free-market levels. Even at the peak during the Vietnam War, steel deliveries for military ordnance constituted just two percent of total shipments [45]. In addition, the insurance premium of trade restrictions, represented as their economic cost in terms of lost efficiency, may be quite high and may not even be necessary. In an increasingly competitive global steel market, production is dispersed across multiple countries, including many that are friendly to the United States or even considered allies. It is, therefore, unlikely that steel supplies could be effectively cut off to any particular country in times of crisis.

In April 2017, President Trump instructed the Secretary of the Department of Commerce to start two investigations to determine whether steel and aluminum imports posed a threat to national security [9]. Section 232 of the Trade Expansion Act of 1962 grants the President of the United States authority to adjust the imports of goods or materials from other countries, through tariffs or other means, if it is deemed that the quantity or circumstances surrounding those imports pose a threat to national security. In mid-February 2018, the Department of Commerce released its findings in a confidential report to President Trump. The Department of Commerce concluded that there is indeed a legal rationale for the imposition of steep tariffs on the import of steel and aluminum, as these imports were deemed to jeopardize U.S. national security [46,47]. In particular, Secretary Wilbur Ross recommended in the investigation report either a global tariff of at least 24 percent on steel imports from all countries, a minimum tariff of 53 percent on steel imports from 12 countries, including Brazil, China, Costa Rica, Egypt, India, Malaysia, Republic of Korea, Russia, South Africa, Thailand, Turkey, and Vietnam, or a quota on steel products from all countries equal to 63 percent of each country’s 2017 exports to the U.S [46].

After receiving the department’s affirmative findings and report, President Trump exercised his presidential authority and announced, on March 1st, 2018, the introduction of a tariff rate of 25 percent on steel and 10 percent on aluminum. A week later, he signed two proclamations to legally enact these tariff rates, which would become effective starting from March 22nd, 2018. Initially, the EU, Canada, Mexico, Australia, Argentina, Brazil, and South Korea were granted a temporary exemption from the order under a carve-out provision [46,47].

The introduction of the tariffs caused quite some political stir and provoked ire from major trading partners. In response to these U.S. tariffs, several countries, including China, the European Union, and Canada, have voiced their concerns and declared that they would start imposing retaliatory tariffs on a wide range of U.S. exports if they were subject to the new U.S. tariffs. On April 30th, 2018, the tariff schedule was extended as the U.S. government announced that the steel and aluminum tariff exemption for the European Union, Canada, and Mexico would be revoked, and these countries would also become subject to the tariff starting from June 1st, 2018. In contrast, Argentina and Australia successfully negotiated a permanent exemption from the steel tariff [46,47].

From mid-June 2018 onwards, many countries actually began to take retaliation measures to impede the exports of goods from the U.S. For instance, on June 22nd, 2018, the EU announced that it would start imposing duties from July on $3.4 billion worth of U.S. steel, agricultural, and other products, while Mexico vowed to impose duties on all imports from the U.S. Canada’s government announced it would impose tariffs on as much as $12.8 billion of U.S. steel, aluminum, dairy, and other products. Moreover, China accused the U.S. of starting a trade war and, on July 6th, imposed retaliatory tariffs equivalent to $34 billion, the equal amount as the expected revenues accruing from the steel and aluminum tariffs on Chinese items. India planned to recoup trade penalties of $241 million on $1.2 billion worth of Indian steel and aluminum. At the same time, the EU, China, Turkey, Mexico, Switzerland, and Canada accused the U.S. of unfair trade practices, and all filed WTO complaints against the U.S. import tariffs. In contrast, the tariff negotiations in North America were relatively more successful, with the U.S. lifting the steel and aluminum tariffs on Canada and Mexico on May 20th, 2019, joining Australia and Argentina as the only nations being exempted from the tariff [46].

Not all threats were eventually converted into retaliatory actions. For instance, Japan and South Korea, both important U.S. allies and trading partners, engaged in diplomatic efforts to address the issue rather than materializing the threats expressed earlier. In 2021, the U.S. and the European Union signed an agreement to lift the barriers on steel and aluminum trade. For the other countries, the tariffs remained in place. However, the Biden administration has expressed on various occasions that the tariffs are subject to review in the near future. In particular, one serious option they are considering is to lift the general tariff under a certain volume and replace it with bilateral quotas. In 2022, several WTO dispute settlement panels have ruled that the tariffs contravene WTO trading rules [48]. The panels recommended that the United States should bring its trade policy into conformity with its obligations under the WTO agreements. However, the United States dismissed the rulings as flawed and declared that it would continue to impose tariffs on most countries. It has now appealed the rulings even though its own actions have impeded the ability of the WTO Appellate Body to hear any appeals. For instance, the U.S. still refuses to appoint a new judge into the WTO ruling council.

Protectionist policies usually trigger a chain reaction. In this case, the introduction of the import tariff on steel and aluminum carried at least three important consequences for the defense-related industry in the U.S. (see, e.g., [49–51]). First, over the last decade, roughly a quarter of U.S. steel demand has been met by steel imports. In response to a tariff, steel imports from target countries were likely to be replaced by less competitive domestic producers. Advocates for a tariff increase emphasized the need to shield the domestic steel industry with high tariffs on imported steel to avoid reliance on foreign steel imports for building weapon systems. They feared that the major steel-producing countries might exploit their monopoly power to cut the steel supply to the United States during a national security crisis. The question, however, is whether this fear was justified as the U.S. defense industry uses virtually no steel or aluminum imported from foreign countries as a result of, for instance, the Buy America Act and other domestic content requirements for specialty metals. Despite the U.S. defense industry being one of the largest users of U.S. steel, it only accounts for a small part of the total steel production. Looking ahead, based on expectations of increased production rates of weapon systems, the U.S. Department of Defense estimated that it would need approximately five percent of domestic steel production [52]. This relatively low market share offers little indication of a near-term supply shortage, and the trends in steel production and import penetration identified in the Commerce Department’s investigation do not suggest that steel requirements are heading toward any significant danger of going unfilled.

Notwithstanding the fact that the U.S. defense industry predominantly buys steel and aluminum from domestic suppliers, the tariffs are expected to have enabled those suppliers to raise prices as they are less hindered by foreign competition. Price increases should find their way into the supply chain as firms deplete their stockpiles. After all, the fundamental purpose of the tariffs was to allow domestic steel producers to raise their prices. This is particularly crucial for the production costs of many heavy defense items, such as navy ships or fighter jets. Cost estimates of these items indicate that over 30 percent of the total production costs are determined by the use of steel and aluminum (see, e.g., [53]). Consequently, the U.S. defense industry would bear higher prices for these metals for as long as the tariffs are in place. The empirical evidence reported by Amiti et al. [54] demonstrated that there was almost a complete passthrough of the tariffs into domestic prices and that the total incidence of the tariff falls mainly on domestic consumers.

Second, the U.S. tariff hikes were likely to strain U.S.-foreign relations as other countries may view the United States as a less reliable trade partner (see, e.g., [28–33]). Target countries, including key allies, retaliated against the coercive trade measures by procuring more key equipment from other nations. This may have caused a fall in the export demand of the U.S. defense industry. Meanwhile, these countries may follow the same tariff logic by taxing intermediate products or other raw materials needed in the U.S. defense industry or require higher percentages of content from their domestic suppliers in U.S. systems. The escalation of the tariff dispute might, in particular, be bad news since the aerospace and defense industries together generate the largest trade surplus of any U.S. manufacturing sector. In total, the defense industry produces about ten percent of all U.S. exports in goods [55]. These foreign contracts are crucial as the domestic demand often falls short to absorb the production capacities of firms and cover the high fixed R&D costs. The essential question is only whether the import demand for U.S. defense items is relatively elastic or inelastic. Due to economies of scale and comparative technological advantages, the U.S. defense industry wields some substantial market power [56]. This limits the substitution possibilities for trading partners to rely on other arms-exporting countries, making retaliation actions directly against the U.S. defense industry less likely to be successful. However, it is possible that trading partners target sectors that are within the supply chain of the U.S. defense industry, such as raw materials or technology. Indirectly, these actions will raise the costs of defense companies in the U.S. In the latter situation, the question remains if the U.S. defense industry is able to pass the higher unit production costs on to the buyers.

Third, the import tariff may have caused an excess supply in the global steel and aluminum market. This surplus may have pushed down the global price of steel and aluminum and may have created a two-tiered market as more cheap metals were available in markets outside the United States. In return, the fall in the prices of steel and aluminum might have provided foreign competitors with a cost advantage. However, according to Amati et al. [54], foreign exporters of steel and aluminum were reluctant to lower their prices to offset the additional cost of the tariffs to their consumers. Despite this, steel and aluminum prices in the United States have remained high since mid-2018. Particularly, steel prices in the U.S. were already well above almost any country in the period before the tariff, with a fifty percent premium over Europe and approximately eighty percent over China [55,57].

In contrast to these arguments, it can be asserted that the stock market return of U.S. defense companies may not be significantly affected by the tariff. This claim rests on two arguments. First, defense firms usually maintain a large inventory of raw materials to fulfill long-term supply contracts. As a result, in the short term, the defense industry is relatively insulated from price fluctuations. However, over the long-term, as inventory levels deplete and existing contracts expire, companies may disclose vulnerability to price risk [58,59]. Second, a significant part of the concluded defense contracts is subject to "cost-plus", enabling defense companies to pass-through the increased raw material prices to the end-user. Compared to other economic sectors, this would limit the exposure of the profitability of the defense industry to input price shocks [59,60].

Based on these considerations, one could argue that the sales revenue would have declined, and the unit production cost would have risen in the U.S. defense industry after the introduction of the steel and aluminum tariff hikes. Meanwhile, retaliation measures taken by important trading partners might disrupt the supply chain, thereby raising the costs even further, or cause a fall in the export demand. Consequently, the profitability and cash flow of defense firms would face some downward pressure, ultimately reducing the market value of these companies [61]. However, it is well known that changes in expectations about introducing a trade barrier or extending it can lead to a shift in investor behavior already long before the restriction is implemented or even agreed upon. Investors’ perceptions will greatly depend on their own risk assessments of the likelihood of these measures and an evaluation of the consequences of tariff impositions for the affected companies. When the risk assessment indicates a shift in the expectations about the firms’ future profits, investors will—in advance of the expected tariff—rearrange their portfolios.

In this specific case, the risk assessment is likely to be built on (i) the steel and aluminum content in the produced items, (ii) the access to alternative metal producers or materials; (iii) the created U.S. trade policy uncertainty in the recent past and the credibility of earlier trade policy threats by President Trump, (iv) the availability of alternatives to U.S.-origin produced military items or contractors, (v) the importance for the U.S. of having stable foreign trade relations with allies, and (vi) the likelihood of retaliation actions by key trading partners that will affect the costs and revenues of U.S. defense companies [62,63]. Hence, based on this assessment, the formal announcement of a tariff imposition may come as no surprise to investors and should, therefore, not lead to any change in asset prices as predicted by the efficient market hypothesis [36,64].

In this context, already during the presidential campaign, Trump repeatedly mentioned his plan to revive the U.S. economy by introducing an economic strategy of "putting America first". One complicating factor is that investors’ perceptions may be impaired by the fact that tariffs can have contrasting effects, making the implications for affected companies unpredictable ex-ante. For instance, Egger and Zhu [32] investigated abnormal share price reactions to tariff announcements and impositions during the U.S.-China trade war in 2018. While the authors find, on average, negative cumulative abnormal returns after most tariff-related events, they also observed that several U.S. sectors were positively affected by China’s retaliation actions. This result suggests that there is a nonuniform effect of trade uncertainty among industries.

## 3. Data

### 3.1 Identifying key tariff-related events

One of the main challenges in exploring the capital market response to the steel and aluminum tariffs is finding a suitable indicator that captures the main events that have created economic uncertainty. While the starting date of the tariff imposition is clear, identifying the remaining critical events associated with this imposition is less clear. To come up with a list of key events, I apply a three-stage approach (see also [36,65]).

**Fig 1:**
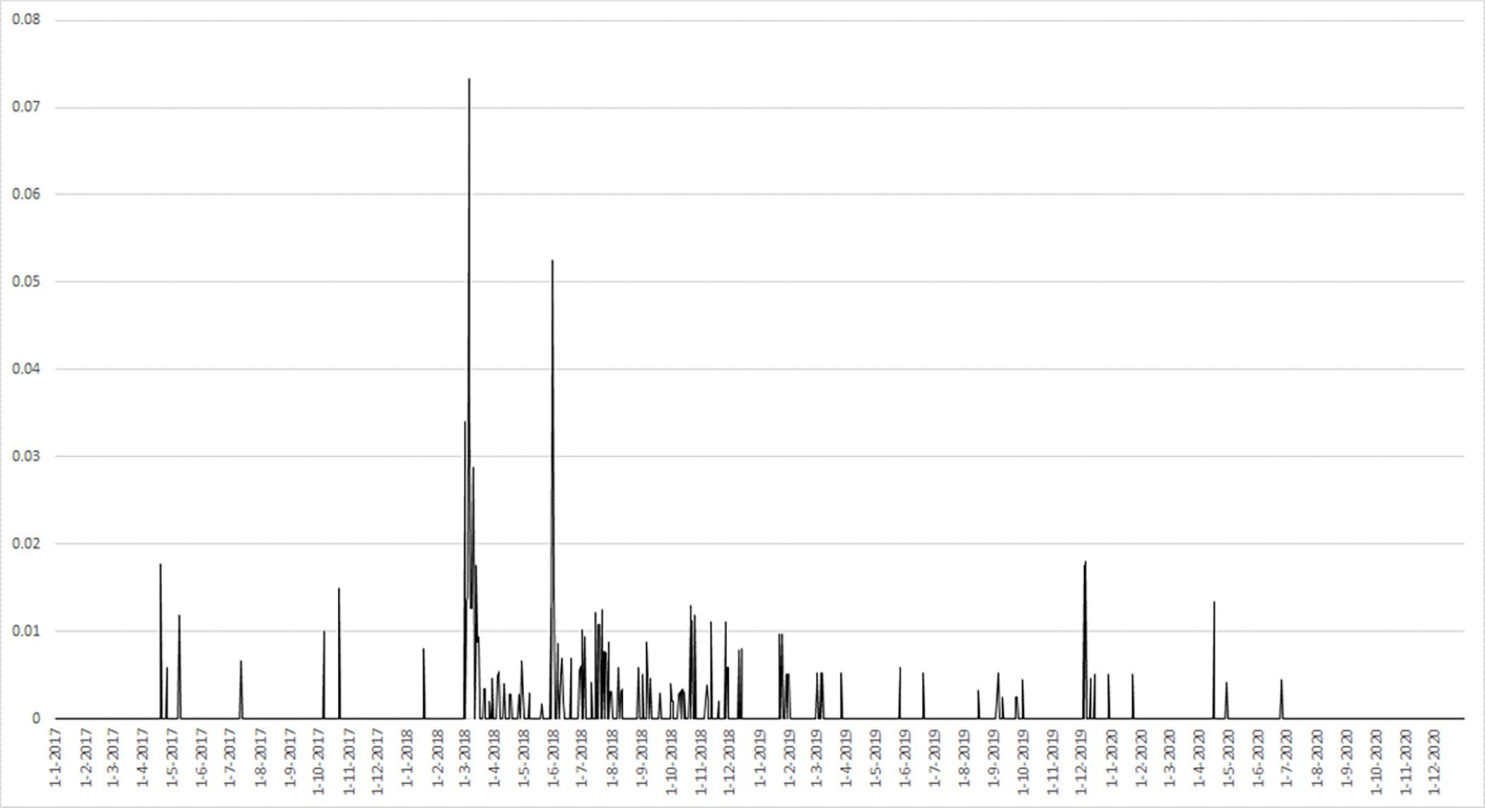
Distribution of newspaper articles.

In the first step, I separate key event dates from more regular days. This selection process is based on the news coverage of tariff-related events and statements using the information provided by the LexisNexis Academic database. This database collects the world’s most reputable news. In particular, I consider all news items published in major international newspapers, including some specifically devoted to the defense industry (e.g., Aerospace Daily, Air Force Times, Aviation Week’s Homeland Security and Defense, Avionics, Defense Daily, Defense News, Defense and Security, IAC Aerospace and Defense, Inside the Pentagon, etc.) and all the major world financial newspapers (Wall Street Journal, Financial Times, etc.). All news articles are collected containing a joint occurrence of the keywords “tariff “, “Trump”, “Section 232”, “trade expansion act”, “steel”, and “aluminum” over the period January 2017 and December 2020. The idea behind this approach is based on the assumption that when the trade dispute between the U.S. and the rest of the world on the steel and aluminum import tariffs starts to escalate, it will be covered more extensively in newspaper articles and read by investors to obtain information. Increased attention to trade policy concerns illustrates the uncertainty of stock market investors regarding how to perceive the recent trade dispute. In particular, investors update their expectations about the economy more frequently during periods of high news coverage than during periods of low news activities [66]. Fig 1 shows the density of tariff-related news articles during the period of analysis (y-axis). The graph indicates various peaks in the frequency related to some critical events, including the announcement of the investigation by the Secretary of Commerce, the release date of the investigation report, the actual imposition of the tariff, and the granting and withdrawal of several tariff exemptions to certain countries.

**Fig 2:**
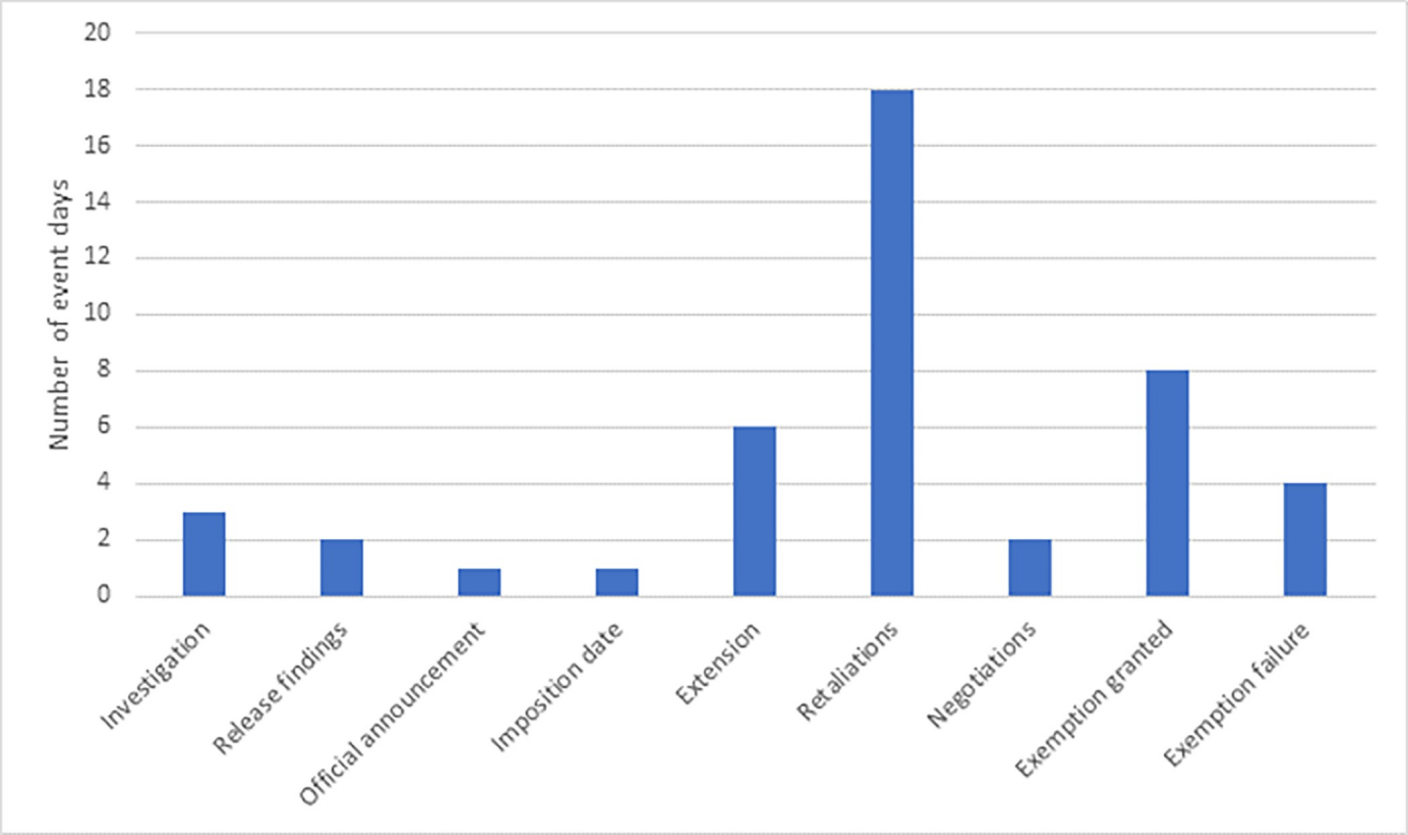
Distribution of key events. Note: “investigation” = Start and progress of the investigation by the Department of Commerce; “Release findings” = Public release of the recommendations from the investigation, including the reaction of President Trump; “Official announcement” = Official announcement of the steel and aluminum tariffs; “imposition date” = Formal initial imposition date of the steel and aluminum tariffs; “extension” = Formal statements about the extension of the tariff schedule; “retaliation” = Announcement of (potential) retaliation measures taken by trading partners; “negotiations” = Start of tariff exemption negotiations; “exemption granted” = Granting temporarily or permanent tariff exemptions, and “exemption failure” = Withdrawal or refusal of tariff exemption.

In the second step, I filter out the dates on which substantially more articles were published than on other days. A key event day is identified when the sum of news items on a particular day is in the top three percent of the distribution. Following this approach leaves me with 45 significant event days (See the detailed timeline of events in Table A1 in the Appendix in S1 File). In the final step, using the qualitative information taken from the news articles on the selected days, I have classified the event days into nine broad categories, including those related to (1) the start and progress of the investigation by the Department of Commerce; (2) the public release of the recommendations from the investigation, including the reaction of President Trump; (3) the official announcement of the initial steel and aluminum tariffs; (4) the official imposition date of the steel and aluminum tariffs; (5) the formal statements about the extension of the tariff schedule; (6) the announcement of (potential) retaliation measures taken by trading partners; (7) the start of tariff exemption negotiations; (8) the granting temporarily or permanent tariff exemptions, and (9) the withdrawal or refusal of tariff exemption. Fig 2 shows the distribution of the selected event days among the different categories.

The hypothesized direction of the impact of the various event categories on the equity return of the U.S. defense industry is less clear and is likely to differ among the considered categories. Specifically, the key events related to the introduction or extension of the tariff scheme, as well as those related to threats of retaliation by important trading partners, are expected to lead to a negative effect on the stock market return as it raises the risk of any further escalation of the trade dispute. Conversely, events related to exemption negotiations and granting exemptions to important trading countries may generate some more positive sentiment among investors in the U.S. defense industry as the trading relationship between countries could potentially become less strained. This, in turn, might exert some upward pressure on the stock market return.

### 3.2. The U.S. defense industrial base

The U.S. defense industrial base is immense, involving approximately 200,000 companies and a workforce of over 2.5 million people. In 2023, the U.S. Department of Defense (DoD) budget was approximately $816 billion, with a significant portion allocated to procurement and research and development (R&D). Defense contractors such as Lockheed Martin, Boeing, Northrop Grumman, and Raytheon Technologies dominate the industry. The U.S. defense industrial base is composed of a diverse array of entities, including large prime contractors, small and medium-sized enterprises (SMEs), and specialized suppliers. Prime contractors like Lockheed Martin and Boeing are responsible for delivering complete defense systems, while SMEs and suppliers provide critical components and services. The U.S. defense industry plays a dominant role in the international defense market, accounting for about two-thirds of the global sales revenues and export value [67]. However, the U.S. defense industry also faces competition from emerging defense industries in countries like China.

The first step in conducting the event study involves defining the sample of U.S. defense-related firms. To ensure a representative sample and to avoid any selection bias, I started with the various editions of the “World Top 100 Defense Firms” reported by the Defense News Media Group and the “Top 100 arms-producing and military services companies” published by the Stockholm International Peace Research Institute (SIPRI). These rankings are based on annual defense sales. This data is complemented by using the Google Finance list of defense-related stocks. To obtain a coherent sample for my study, I followed Capelle-Blancard and Couderc [37] by applying some exclusion criteria. Since my focus is on publicly listed companies, I exclude: (1) fully and partly state-owned firms; (2) family-owned firms; (3) firms with one dominant shareholder or with a low free float rate and (4) firms with defense revenue below ten percent of their total revenue.

In the next step, I have collected the daily stock prices of the selected firm. This data is primarily taken from Thomson Datastream, Bloomberg, and Yahoo Finance. After excluding the firms for which no data was available, I retained a sample of 66 defense-related firms that constitute my sample (the complete list of companies is shown in Table A2 in the Appendix in S1 File). It is important to note that since my sample is partly based on various issues on the defense Top 100 rankings reported by SIPRI, it may have a potential bias towards larger companies. In particular, small and medium enterprises (SMEs), especially start-ups and scale-ups, are generally privately owned. However, these companies play an important role in the R&D activities taking place in the defense industry.

To summarize, the companies in my sample account for up to 80 percent of the sales revenues generated in this industry. My sample of defense companies’ average daily stock market return is +7 basis points (bps), with a median of +13 bps and a standard deviation of 1.71 bps. On average, approximately 60 percent of the sales revenues from the included firms can be attributed to military sales, while these firms have a median work force of about 15 thousand employees.

### 3.3. Abnormal returns

The event study method is based on the market model of stock valuation. It assumes that the stock price reflects the time and risk-adjusted discounted present value of all future cash flows that are expected to accrue to the holder of that stock. In order to isolate stock market reactions to the events related to the steel and aluminum tariff war between the U.S. and the rest of the world, I control for market co-movement and exclude potentially confounding events. In particular, I explore the impact of tariff-related events on the abnormal return in the U.S. defense industry. In finance, the abnormal return is defined as the difference between the actual return of a security and the expected return. Abnormal returns are typically triggered by events that influence the market value of a firm, but have not yet been priced by the market [40–42].

The next step in the analysis is to estimate the expected return of the stock during a specific time window, assuming that an event never took place. To compute this expected return, I estimate an asset pricing model that includes the risk-free rate, the market return, and the so-called Fama-French factors. In particular, the following empirical model is estimated using the OLS estimator.

$$r_{it}=E[r_{it}]+e_{it}$$

$$E[r_{it}]=\beta_{0i}+\beta_{1}(r_{t}^{M}-r_{t}^{F})+\beta_{2}{SMB}_{t}+\beta_{3}{HML}_{t}+\beta_{4}{UMD}_{t}$$ (1)

Where *r*_*it*_ is the stock market return of defense company *i* at day *t*, The daily return is measured through closing prices. The variable *r*^*F*^ is the risk-free rate based on the ten-year U.S. government bond rate, *r*^*M*^ is the market return measured by the return of the S&P 500 index. The variable *SMB* (small minus big) is the difference between the daily returns of the small and big firms’ portfolios, *HML* (high minus low) is the difference between the daily returns of high book-to-market and low book-to-market firms’ portfolios, and *UMD* (up minus down) is the momentum factor computed as the daily return differential between a portfolio of winners and a portfolio of losers. These latter Fama–French factors are taken for the U.S. market and obtained from the homepage of Kenneth French at Dartmouth College. Finally, *β*_i0_ is a company-specific effect to control for average company productivity or size [32], while *e*_*it*_ is the residual that captures unanticipated random events.

In line with Armitage [68], I estimate Eq (1) applying a time window of 250 trading days, i.e., [- 260; - 11], where *T* = 0 is the event day for my analysis, i.e., the official announcement by President Trump to start an investigation by the Department of Commerce. The length of 250 trading days ensures a greater precision of regression coefficient estimates than shorter estimation windows. However, a longer window increases the likelihood that it contains information caused by other events [69–71].

The final step in computing the abnormal return is subtracting the expected return from the realized return observed during the event period. Accordingly, abnormal stock returns (*AR*) are computed as follows.


$${AR}_{it}=r_{it}-E[r_{it}]$$ (2)

The cumulative abnormal return (CAR) for firm *i* during the event window [*τ*_1_; *τ*_2_] surrounding a single event day *t* = 0, is given by

$$C{AR}_{i,[\tau_{1};\tau_{2}]}=\sum_{t=\tau_{1}}^{\tau_{2}}{AR}_{it}$$ (3)

In order to analyze observations across *N* firms, I define the cumulative average abnormal return (CAAR) as

$$C{AAR}_{\left[\tau_{1};\tau_{2}\right]}=\frac{1}{N}\sum_{i=1}^{N}C{AR}_{i,[\tau_{1};\tau_{2}]}$$ (4)

**Fig 3:**
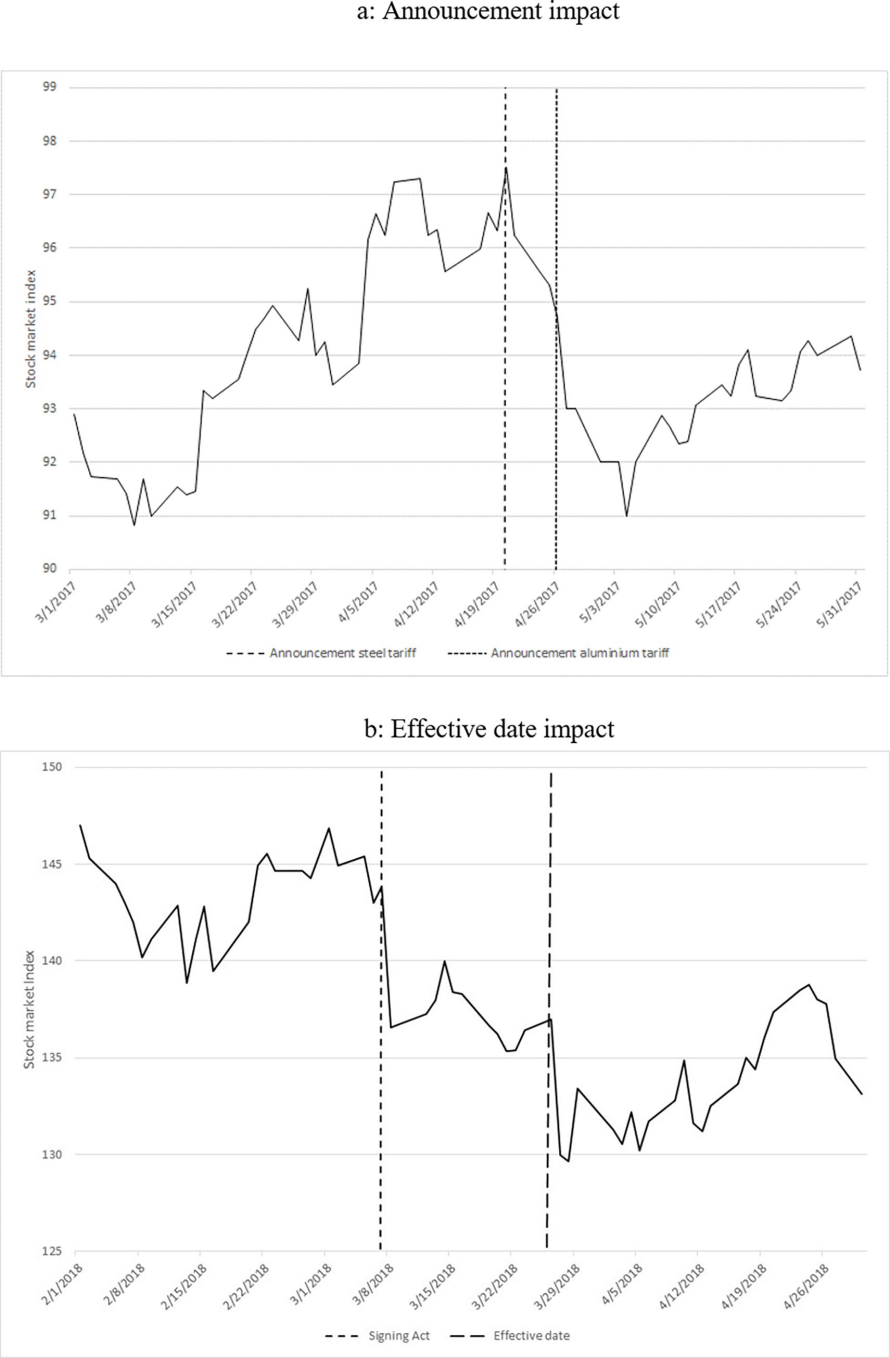
a: Announcement impact. b: Effective date impact.

**Fig 4:**
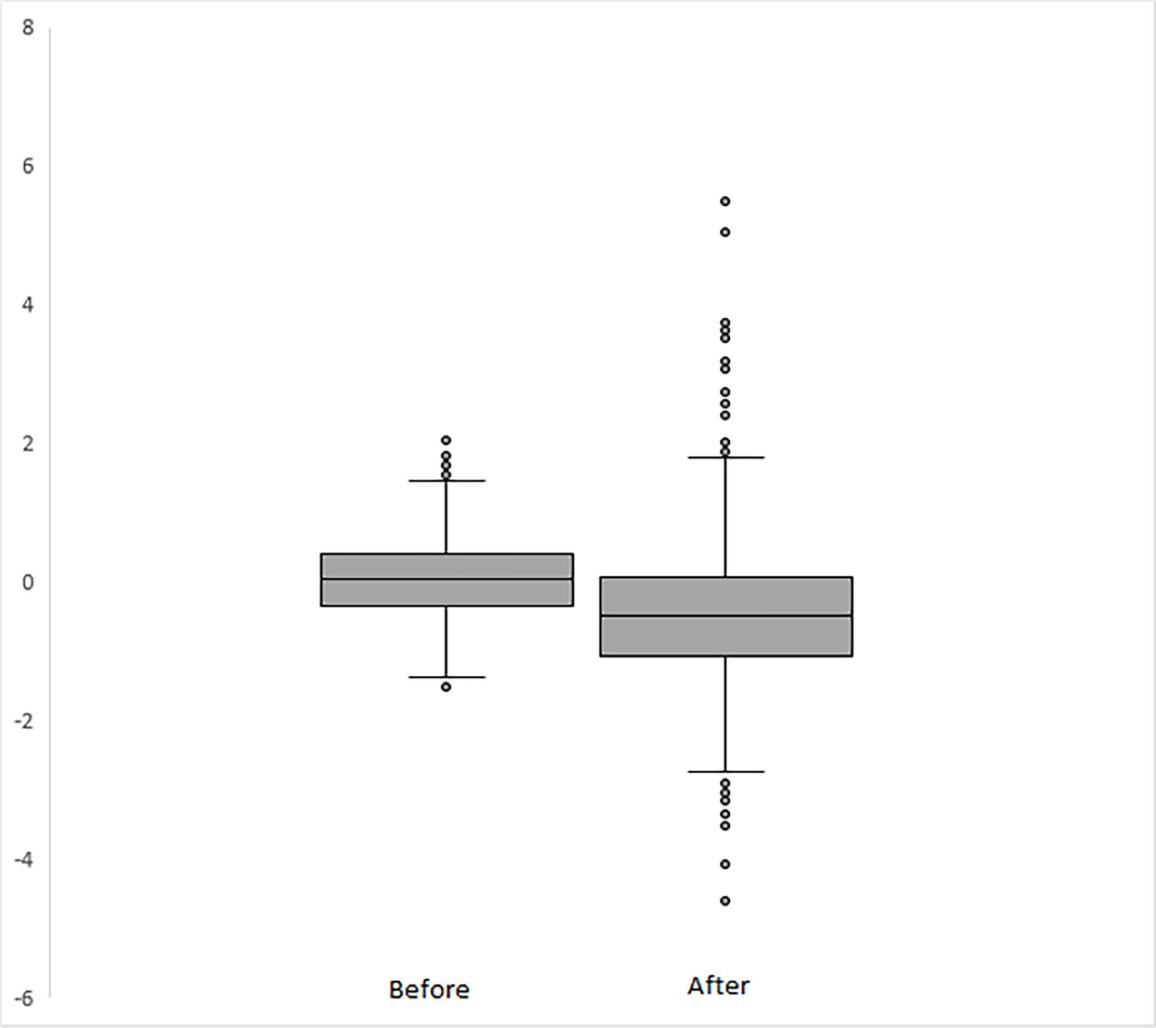
Boxplot of the abnormal returns of U.S. defense companies. Note: “before” indicates the boxplot based on the observations before the official announcement of the tariff scheme by President Trump in March 2018, while “after” reports the boxplot based on the observations after this formal announcement.

As a preliminary analysis, I explore graphically the impact of the introduction of the tariff scheme. Fig 3A and 3B illustrate the impact of the ordering of the investigation, the official announcement of the tariff scheme and the effective imposition date. The graphs show a clear drop in the abnormal return after these events. Additionally, the graph in Fig 4 shows the boxplot of the daily average abnormal returns throughout the period of my analysis. It is evident that the volatility of abnormal returns amplified following President Trump’s announcement of the investigation. More specifically, before the announcement of the investigation, the standard deviation of the abnormal returns was about 0.8 and approximately 1.2 afterward. This difference is statistically significant and indicates that the uncertainty among investors in the U.S. defense industry has increased after the announcement. Nevertheless, it is essential to note that this graphical analysis serves only as an indication, as unobserved company heterogeneity and other potentially confounding variables have not been accounted for.

## 4. (Non)parametric analysis

In order to test whether the CAARs are significantly different from zero after a tariff-related event, I employ two parametric tests—namely, the Adjusted-Patell test and the BMP test—alongside two nonparametric tests: the generalized sign test and the GRANK-T test. All the tests applied share a null hypothesis stating that the cumulative average abnormal return equals zero, with the alternative hypothesis proposing that the CAARs differ from zero.

Given that investors’ expectations might take more than one day to fully adjust to new information, it is necessary to precisely define the event window. In particular, the event window might be asymmetric concerning the day of event realization. The direction of asymmetry depends on the degree of anticipation of the event. If the event is expected, the event window will include several days before it, since the effect of the upcoming event is felt before its actual realization. On the other hand, with unexpected events, the period will comprise several days after the event, because the effect is then manifested in the market. Table 1 presents the results for the cumulative average abnormal stock market return of U.S. defense companies after the different types of events across four intervals, namely [-5; +1], [-1; +1], [-1; 0], and [-1; +5].

**Table 1:** Event analysis.

	Time window							
	[-5;+1]		[-1;+1]		[0;+1]		[-1;+5]	
***Start and progress investigation***	-0.0013		-0.0028		-0.0030		-0.0014	
Adjusted Patell test	-1.827	*	-3.520	***	-4.609	***	-1.938	*
BMP test	-2.330	**	-2.159	**	-3.951	***	-1.820	*
General sign test	-2.291	**	-2.283	**	-3.628	***	-0.944	
GRANK test	-1.978	**	-2.223	**	-4.548	***	-0.975	
***Public release investigation report***	-0.0003		-0.0005		-0.0005		-0.0002	
Adjusted Patell test	-0.913		-1.379		-3.672	***	-1.452	
BMP test	-1.543		-0.896		-5.054	***	-0.918	
General sign test	-1.337		-1.047		-5.060	***	-1.019	
GRANK test	-0.922		-1.397		-4.284	***	-1.239	
***Official announcement tariff***	-0.0008		-0.0015		-0.0017		-0.0007	
Adjusted Patell test	-2.130	**	-4.632	***	-4.980	***	-1.679	*
BMP test	-2.340	**	-3.705	***	-3.848	***	-1.747	*
General sign test	-1.811	*	-3.423	***	-4.382	***	-1.199	
GRANK test	-2.131	**	-4.093	***	-3.922	***	-1.718	*
***Formal imposition date***	-0.0038		-0.0061		-0.0062		-0.0030	
Adjusted Patell test	-2.000	**	-2.169	**	-2.293	**	-1.930	*
BMP test	-1.724	*	-1.698	*	-2.000	**	-1.839	*
General sign test	-0.838		-1.868	*	-2.358	**	-0.976	
GRANK test	-1.209		-2.112	**	-2.114	**	-0.812	
***Statements about extending tariff***	-0.0012		-0.0017		-0.0021		-0.0010	
Adjusted Patell test	-1.521		-1.001		-0.825		-1.337	
BMP test	-1.080		-1.331		-0.995		-0.941	
General sign test	-1.366		-0.890		-1.158		-0.955	
GRANK test	-1.543		-1.037		-1.227		-1.341	
***Announcing retaliation actions***	-0.0006		-0.0014		-0.0013		-0.0005	
Adjusted Patell test	-1.519		-1.983	**	-1.981	**	-1.028	
BMP test	-0.940		-1.704	*	-2.289	**	-0.862	
General sign test	-0.906		-2.001	**	-1.836	*	-1.050	
GRANK test	-1.034		-1.838	*	-2.003	**	-1.245	
***Start exemption negotiations***	0.0007		0.0013		0.0014		0.0008	
Adjusted Patell test	1.368		1.997	**	2.014	**	0.851	
BMP test	0.975		1.869	*	1.850	*	1.528	
General sign test	1.474		1.902	*	1.852	*	1.455	
GRANK test	1.131		1.812	*	1.791	*	1.039	
***Exemption granted***	0.0005		0.0010		0.0009		0.0004	
Adjusted Patell test	1.346		1.366		0.960		1.173	
BMP test	1.551		1.597		1.259		1.245	
General sign test	0.996		1.320		1.266		1.196	
GRANK test	0.979		1.092		1.585		1.240	
***Exemption withdrawn***	-0.0005		-0.0011		-0.0012		-0.0005	
Adjusted Patell test	-1.255		-1.577		-0.825		-1.542	
BMP test	-1.331		-1.302		-0.802		-1.592	
General sign test	-1.526		-0.896		-0.884		-1.055	
GRANK test	-1.509		-1.480		-1.532		-1.371	

**/* indicates significance at respectively 10 and 5 percent level.

Based on the results of the parametric and nonparametric tests reported in Table 1, several conclusions can be drawn. First, events days related to the ordering by President Trump to initiate an investigation based on Section 232 of the Trade Expansion Act by the Department of Commerce, as well as, the progress of this investigation, already led to a drop in the stock market prices. This finding implies that even before the release of the findings, these events created economic uncertainty. Investors’ perceptions regarding this investigation were shaped by their expectations and available information. Investors speculated that Trump would start introducing trade barriers that were detrimental to the U.S. defense industry, regardless of the outcome of the investigation [54,72–74].

Although investors have anticipated the possible introduction of trade restrictions, they did not perceive the full extent of their impact until the official announcement of the tariffs, which further exacerbated the negative sentiment. This result aligns with the empirical evidence of Bianconi et al. [28], suggesting that stock market investors tend to underestimate the consequences of trade policy changes due to their unpredictable nature for exposed companies. The overall assessment of the tariff announcement by investors hinges on the estimated economic ramifications of a tariff—higher input costs, lower profit margins, reduced trading volumes, disrupted supply chains, and worsening competitiveness. As a result, investors expected the market value of U.S. defense companies to fall.

Moreover, it turns out that the threat or actual imposition of retaliatory measures by other countries exerts a significant negative impact on investor sentiment. Investors in the U.S. defense industry interpreted the retaliatory message as a sign that the tariff dispute might escalate into a full-fledged trade war, which would not only impede defense sales revenues, but also escalate costs of intermediate goods or materials crucial for production. In response, investors adjusted their investment behavior using this new information. In turn, event days linked to the commencement of tariff negotiations between the U.S. and important trading partners yield a positive effect on the abnormal returns of U.S. defense companies. These negotiations were primarily aimed at enhancing trade relations between the U.S. and the participating countries. This effect is particularly pronounced when exemptions are granted, as this further reinforces the positive impact.

Additionally, I find any significant effect of statements regarding the extension of the tariff or about the status of an exemption. The absence of a significant effect of certain event categories can be attributed to four potential reasons (see also [75]). First, investors may not have reacted to these specific events as they expected that the profitability of U.S. defense companies would be unaffected by them. Second, the reaction of investors might have occurred outside the chosen time window. To address this concern, I have estimated the main model using different event windows, as indicated above. Upon comparing the results across these different time windows, it appears that both positive and negative market reactions are more pronounced and statistically significant closer to the event days. This strongly supports the notion that capital markets react highly rationally in light of the trade dispute, with necessary adjustments largely made on the event day itself. This reinforces the belief in the efficiency of capital markets.

Third, the sample of defense firms on which the estimation is based is quite heterogeneous. While some companies may have been negatively affected by these import tariffs due to their reliance on steel and aluminum or suffered from retaliatory actions by foreign countries, others may not have been affected or even benefitted. Arguably, aggregating all these firms together in one estimate could lead to a zero net effect, as the negative effects found might be offset by positive ones. To address this issue in more detail, I differentiate in the next section between different types of defense companies based on several firm characteristics. Finally, investors may differ in the extent to which they have information about a particular company or industry. On the one hand, some investors may have actively gathered detailed information about a company or sector to monitor it closely. These investors likely had a much clearer view of the potential consequences of the tariff-related events on the profitability of that particular firm. On the other hand, most investors are likely to have only limited information. It is possible that these two types of investors differ in their responses to tariff-related events. Therefore, combining the responses of these investors may diminish the significance of the effect observed in these events, as no single reaction dominates.

## 5. Panel regression analysis

### 5.1 Model specification

One drawback of the previous parametric and nonparametric tests is that they are only suggestive, as they do not account for other confounding variables that may also influence abnormal returns. Besides, as indicated in the timeline of events listed in Table A1 in the Appendix in S1 File, several key events identified in my timeline occurred almost on the same date, thereby creating some simultaneity and contagion concerns. Especially the extension of the tariff schedule or tariff exemption negotiations failures are promptly followed by retaliation threats. Therefore, it is important to isolate the individual impact of each event category. To estimate the impact of tariff-related key events on the cumulative abnormal stock market return of U.S. defense companies, I employ a regression model using an impulse response function. More formally, I use the following general specification.

$${AR}_{it}=\alpha_{i}+\gamma x_{it-n}+\sum_{k=0}^{7}\theta_{j}{Event}_{t-k}^{j}+\delta_{month-year}+\delta_{day}+\delta_{t}+\varepsilon_{it}$$ (5)

Where *AR*_*t*_ is the daily abnormal stock return of a U.S. defense company *i* at day *t*, as given in Eq (2). The parameter *α*_*i*_ is a company-specific intercept and controls for time-invariant company characteristics. Since I use a relative short time window of three years, many company-specific variables have a rather static nature in my dataset. For instance, defense contracts are spread over multiple years and often tied to additional long-term obligations such as service and maintenance. Likewise, large-scale R&D investment programmes have usually a put through time of many years [76]. The vector **x** includes several lagged economic and firm-specific control variables. The optimal number of lags *n* on the control variables is determined by the Schwarz Bayesian Information Criterion (SBC) to avoid any simultaneity and endogeneity concerns. The vector **Event** represents a series of dummy variables taking the value one when a particular day is identified as a key event date in a specific tariff event category *j* and zero otherwise. To explore the persistence effect of the events, I have included the event measures with up to 7 lags. The (non)parametric tests already revealed that the price discovery of U.S. defense stocks is relatively efficient. However, exploring only the event day might create some timing concerns since information may be released after the closing of the trading system. The hypothesis tested in this study is that the parameter *θ*_*j*_ in Eq (5) is statistically significantly different from zero as investors react to new tariff-related information that could have important implications for the future cost of production and the likelihood of retaliatory actions of trading partners in the near future. However, as already discussed above, the direction is not immediately clear as some key events will adversely affect investors’ sentiment, while others will improve it.

In the vector of control variables, I consider variables suggested by previous studies explaining equity returns. These covariates are required to avoid an omitted variable bias. First, the volatility index (VIX) of the Chicago Board Options Exchange (CBOE) is added as a proxy for financial market uncertainty. The VIX measures the implied volatility from option contracts on the Standard and Poor’s 500 index and can be interpreted as a forward-looking indicator of global risk aversion. Furthermore, the monthly change in the real exchange rate index is included. A less competitive exchange rate may reduce the export demand for U.S. defense items. The real exchange rate index is based on the exchange rate between the U.S. dollar and a basket of the most important international currencies, including the Euro, the Japanese Yen, and the British Pound Sterling.

Moreover, the Covid-19 pandemic substantially increased uncertainty among investors in the U.S. defense industry due to major supply chain disruptions, including the shortage of chips needed in production and the shutdown of production lines and factories. To control for the Covid-19 pandemic, I include a dummy variable taking the value one after January 31st, 2020, when President Donald Trump declared the outbreak as a public health emergency.

During the period of my analysis, another important geopolitical shock occurred that is likely to have affected primarily the equity return in the U.S. defense industry. In October 2018, Jamal Khashoggi, a journalist, and critic of the Saudi Arabian regime, disappeared after a visit to the Saudi Arabian consulate in Turkey. After this incident, it was unclear whether a major arms deal that was signed between the United States and Saudi Arabia would still get approved by Congress [36]. To capture this event, I include a dummy variable taking the value one in the post-event period.

Additionally, I added three company-specific control variables. First, the size of a company, as measured by its asset size (taken in logarithms). Second, the daily volume of trade of a specific stock, which reflects its degree of liquidity. Lastly, a dummy variable taking the value one when companies are being involved in M&A activities in a particular year (Table A4 in the Appendix in S1 File provides the descriptive statistics of the variables used). Moreover, day-of-the-week fixed effects (*δ*_day_) are added to control for trading day effects, while *δ*_month-year_ captures a month-year effect. Finally, *δ*_*t*_ represents a linear time trend to further address the general trade policy uncertainty caused by the decisions of President Trump. The model given in Eq (5) is estimated using Feasible Generalized Least Squares (FGLS) estimation with heteroscedastic error terms.

### 5.2 Basic estimation results

The results of my regression analysis are presented in Table 2. The table shows the results from the impulse response of the first three trading days together with the cumulative abnormal return over a week that can be attributed to the specific tariff-related events. Following the rule of thumb suggested by Davidson and MacKinnon [77], I apply the bootstrap procedure with 1,500 replicators to obtain robust standard errors. More specifically, the standard errors are clustered at the company level and computed using the cross-section variance of abnormal returns to account for variance inflation on event days. To begin, I estimate a baseline panel model in column (1). Based on the estimation results of this specification, I can draw several conclusions that largely confirm the results from the previous parametric and nonparametric tests reported in Table 1. First, the start of the investigation by the Department of Commerce puts the stock market return of U.S. defense companies already under significant downward pressure. Second, investors did not fully anticipate this, as the official tariff announcement reinforces the negative sentiment. Third, the initiative to start negotiations for tariff exemptions has a positive effect on the equity price of U.S. defense firms. However, the outcome of this bargaining process has no direct influence, as the variables related to exemptions granted or withdrawn have no statistical impact. Finally, the announcement of retaliation measures by certain countries again reduces the stock market return of the considered companies as investors fear a fall in sales revenues or expect higher input prices. However, one important note by comparing the results in Tables 1 and 2 is that in the (non)parametric tests, I try to explain the variation in the Cumulative Average Abnormal Return (CAAR) at the industry level, while in the regression analysis, the dependent variable is based on the Abnormal Returns (AR) at the company level.

**Table 2:** Panel regression analysis—impulse response function.

	(1)		(2)	
***Start and progress investigation***				
t	-0.0028	**	-0.0148	**
	(0.001)		(0.005)	
t + 1	-0.0010		-0.0066	
	(0.002)		(0.007)	
t + 2	-0.0004		-0.0023	
	(0.000)		(0.002)	
t + 3	-0.0001		-0.0005	
	(0.000)		(0.000)	
T = 7	-0.0016	**	-0.0087	**
	(0.001)		(0.003)	
***Public release investigation report***				
t	-0.0005		-0.0010	
	(0.001)		(0.001)	
t + 1	-0.0002		-0.0004	
	(0.000)		(0.000)	
t + 2	-0.0001		-0.0002	
	(0.000)		(0.000)	
t + 3	0.0000		-0.0001	
	(0.000)		(0.000)	
T = 7	-0.0003		-0.0006	
	(0.000)		(0.001)	
***Official announcement tariff***				
t	-0.0019	**	-0.0025	**
	(0.001)		(0.001)	
t + 1	-0.0011		-0.0015	
	(0.002)		(0.001)	
t + 2	-0.0005		-0.0009	
	(0.001)		(0.001)	
t + 3	-0.0003		-0.0006	
	(0.000)		(0.000)	
T = 7	-0.0011	**	-0.0016	**
	(0.000)		(0.000)	
***Formal imposition date***				
t	-0.0061	*	-0.0130	
	(0.003)		(0.025)	
t + 1	-0.0027		-0.0038	
	(0.005)		(0.006)	
t + 2	-0.0010		-0.0027	
	(0.001)		(0.002)	
t + 3	-0.0002		-0.0016	
	(0.000)		(0.002)	
T = 7	-0.0036	*	-0.0073	
	(0.002)		(0.010)	
***Statement about extending tariff***				
t	-0.0020		-0.0042	
	(0.002)		(0.003)	
t + 1	-0.0011		-0.0020	
	(0.001)		(0.002)	
t + 2	-0.0004		-0.0009	
	(0.001)		(0.001)	
t + 3	-0.0002		-0.0003	
	(0.000)		(0.000)	
T = 7	-0.0012		-0.0025	
	(0.001)		(0.003)	
***Announcing retaliation actions***				
t	-0.0013	**	-0.0055	**
	(0.001)		(0.001)	
t + 1	-0.0008		-0.0025	
	(0.001)		(0.002)	
t + 2	-0.0003		-0.0015	
	(0.000)		(0.002)	
t + 3	-0.0001		-0.0009	
	(0.000)		(0.001)	
T = 7	-0.0008	**	-0.0033	**
	(0.000)		(0.001)	
***Start exemption negotiation***				
t	0.0016	*	0.0033	*
	(0.001)		(0.002)	
t + 1	0.0007		0.0019	
	(0.001)		(0.003)	
t + 2	0.0004		0.0011	
	(0.001)		(0.001)	
t + 3	0.0001		0.0003	
	(0.000)		(0.000)	
T = 7	0.0009	*	0.0020	*
	(0.001)		(0.001)	
***Exemption granted***				
t	0.0009		0.0019	
	(0.001)		(0.002)	
t + 1	0.0003		0.0011	
	(0.000)		(0.001)	
t + 2	0.0001		0.0003	
	(0.000)		(0.000)	
t + 3	0.0000		0.0001	
	(0.000)		(0.000)	
T = 7	0.0005		0.0012	
	(0.001)		(0.001)	
***Exemption withdrawn***				
t	-0.0012		-0.0025	
	(0.002)		(0.002)	
t + 1	-0.0005		-0.0008	
	(0.001)		(0.001)	
t + 2	-0.0001		-0.0002	
	(0.000)		(0.000)	
t + 3	0.0000		-0.0001	
	(0.000)		(0.000)	
T = 7	-0.0007		-0.0014	
	(0.001)		(0.002)	
Events	ALL		FIRST-SIGNS	
Sample of firms	ALL		ALL	
Number of observations	61750		61750	

Note: Panel-corrected robust standard errors in parentheses.

*, ** and *** denote statistical significance at the 1%, 5% and 10% level, respectively. All regressions are controlled for heteroskedasticity.

To approximate the overall impact of tariff-related events on the abnormal returns of the considered defense stocks, I multiply the number of events in each category by the specific coefficients found in column (1). It turns out that the tariff-related events together reduced the stock price by about three percent in the period of my analysis. Based on this result, one can argue that the tariff dispute between the U.S. and many other steel-exporting countries was only a minor threat to the business perspectives of the U.S. defense industry. One plausible explanation is that, although these companies had to deal with higher costs, a large of the tariff might be passed on to the buyers of U.S. defense equipment due to the monopoly power of these firms. However, a cautionary note regarding these findings is that I should interpret them more in terms of correlation rather than causation.

It is well argued in the finance literature that geopolitical events work as a learning mechanism and that investors review or adjust their portfolios after such events. This idea implies that the first-time events should be more surprising and may include more relevant information for investors than subsequent events in the same category. If this is true, it suggests that first-time events may have a greater impact on investors’ perceptions and, subsequently, a greater impact on the abnormal returns. To test whether the data supports this idea, I have included in column (2) only the first events from each category. The remaining events are set equal to zero. Comparing the results reported in columns (1) and (2) supports the idea that first-time events have a statistically larger impact on the abnormal return than the remaining events. More than 60 percent of the cumulative effect of the considered events is explained by their first occurrence. This is again in line with the assumption of efficient markets, as price adjustments become less severe if an event occurs repeatedly.

### 5.3 Firm characteristics

In the results presented so far, I have assumed that the impact of the steel and aluminum tariffs is the same for all the U.S. defense companies considered in my sample. However, this assumption is somewhat questionable as the companies included in my analysis are rather diverse. They produce different defense items that differ in their steel and aluminum content. Therefore, their exposure to the tariff events might vary among companies depending on specific firm characteristics (see also [29]). To start, I have split my sample into companies producing items with either a large or low metal content. To classify companies, we follow a two-step approach. In the first step, I categorize companies based on the items they produce. This classification is mainly based on information taken from various versions of the Defense Top 100 reported by the Defense News Media Group, annual company reports and the taxonomy of conventional major arms provided by Levine et al. [78]. In the second step, I classify the produced items in accordance with their steel and aluminium content. This classification is based on a report of KPMG [79] and information of the U.S. Army Corps of Engineers on equipment data and specifications [80]. Companies producing items with a high metal content include, among others, manufacturers of aircraft, ships, armoured vehicles, artillery, and missiles. The companies identified with a low metal demand are mainly in the domain of electronics and communication technology or are involved in service and maintenance. These latter firms are expected to be primarily affected by a second-round effect of the tariff as they are typically hired as subcontractors. If the demand falls for the main contractor, then subsequently, the demand for subcontractors is likely to drop as well. The results in columns (1) and (2) of Table 3 indicate that companies with a low metal demand are initially less directly affected by the possible imposition of the tariff. However, after the imposition, investors in these companies also fear the risk of retaliation from important foreign customers. Nevertheless, one should interpret these results carefully since the split is somewhat arbitrary. Many companies produce a wide range of items that differ in their metal content and, as a result, are affected to different degrees by the steel and aluminum tariffs.

**Table 3:** Panel regression analysis–company characteristics.

	(1)		(2)		(3)		(4)		(5)		(6)	
Start and progress investigation	-0.0068	**	-0.0034	*	-0.0044	**	-0.0025	**	-0.0024	**	-0.0023	*
	(0.002)		(0.002)		(0.002)		(0.001)		(0.001)		(0.001)	
Public release investigation report	-0.0007		-0.0005		-0.0008		-0.0005		-0.0006		-0.0006	
	(0.001)		(0.001)		(0.001)		(0.000)		(0.001)		(0.001)	
Official announcement tariff	-0.0034	**	-0.0016		-0.0017	**	-0.0015	*	-0.0015	**	-0.0011	*
	(0.001)		(0.001)		(0.001)		(0.001)		(0.000)		(0.001)	
Formal imposition date	-0.0157		-0.0076		-0.0117	*	-0.0069	*	-0.0057		-0.0091	
	(0.014)		(0.014)		(0.006)		(0.004)		(0.007)		(0.012)	
Statement about extending tariff	-0.0042		-0.0013		-0.0018		-0.0024		-0.0025		-0.0012	
	(0.004)		(0.001)		(0.002)		(0.002)		(0.002)		(0.002)	
Announcing retaliation actions	-0.0029	**	-0.0010	**	-0.0011	*	-0.0011	**	-0.0013	**	-0.0015	**
	(0.001)		(0.000)		(0.001)		(0.000)		(0.000)		(0.001)	
Start exemption negotiation	0.0028		0.0011		0.0019		0.0013	*	0.0019		0.0017	
	(0.002)		(0.001)		(0.003)		(0.001)		(0.001)		(0.001)	
Exemption granted	0.0020		0.0005		0.0013		0.0007		0.0010		0.0008	
	(0.001)		(0.000)		(0.001)		(0.001)		(0.001)		(0.001)	
Exemption withdrawn	-0.0027		-0.0011		-0.0019		-0.0016		-0.0014		-0.0006	
	(0.002)		(0.001)		(0.002)		(0.001)		(0.001)		(0.001)	
Accumulated impact	-6.2%		-2.4%		-2.7%		-2.2%		-2.4%		-2.6%	
Events	ALL		ALL		ALL		ALL		ALL		ALL	
Sample of firms	Major steel consumers		Minor steel consumers		Military dominated		Civil dominated		Small companies		large companies	

Note: Panel-corrected robust standard errors in parentheses.

*, ** and *** denote statistical significance at the 1%, 5% and 10% level, respectively. All regressions are controlled for heteroskedasticity.

On a related note, the economics literature recognizes the extent of diversification as a kind of implicit insurance against uncertainty in trade policies [81,82]. When applying this logic to the case of how the import tariffs will affect the profitability of the defense industry, it is expected that more diversified firms will be better able to deal with this uncertainty. As a first test on diversification, I explore whether the import tariff has the same effect on firms that produce primarily military items and companies that also produce civil or dual-use goods. The expectation is that these latter companies can better diversify their business activities among markets and, therefore, suffer less from trade policy uncertainty. However, according to the results presented in columns (3) and (4) of Table 3, it turns out that the reverse is actually true, as dual-use companies suffer more from the key events, especially the ones related to retaliation measures. One possible explanation is that dual-use companies must deal with a double effect—higher costs of materials and lower expected exports after retaliation actions as the international competition on the civilian market is typically more intensive due to the availability of multiple alternatives. In turn, U.S. defense companies that produce primarily military items usually have a better market position due to the dominance of the U.S. defense industry.

Moreover, this diversification effect is typically related to the size of a company (i.e., [83]). Larger companies typically produce a broader array of military goods, often targeting various markets or customer segments. In case of shocks, it is easier shifting between different production lines. In contrast, smaller companies are less able to diversify their business activities or markets as they produce only a limited range of goods or services. To examine this latter issue in more detail, I have split my company sample in the final columns of Table 3 into two equal size subsamples based on the median asset size (around 12 billion U.S. dollars). Surprisingly, the difference in impact in most event categories’ is statistically negligible, arguing that the impact is almost the same between large and small companies. However, one critical remark about these findings is that the sample will likely be biased toward larger firms as SMEs are missing since they are usually privately owned.

### 5.4 Contagion between tariff schemes

During my period of analysis, besides the Section 232 tariffs, also the Section 301 tariffs from the Trade Act of 1974 were introduced. The Trade Act authorizes the U.S. president to take all appropriate action, including tariff-based and non-tariff-based retaliation, to obtain the removal of any act, policy, or practice of a foreign government that violates an international trade agreement or is unjustified, unreasonable, or discriminatory, and that burdens or restricts U.S. commerce [84].

On March 22, 2018, Trump signed a memorandum instructing the United States Trade Representative to apply tariffs of $50 billion on Chinese goods. The tariffs targeted a wide range of imports, including washing machines, solar panels, food, automobiles, sports equipment, electronics, and chemicals. The imposition of these tariffs was justified by alleged Chinese theft of U.S. intellectual property and unfair trade practices. In response to the Section 301 tariffs, the Ministry of Commerce of the People’s Republic of China announced to implement its own tariffs on a large number of U.S. products, such as fruit, wine, and pork [84].

These Section 301 tariffs are expected not to affect the economic performance of the U.S. defense industry. This expectation is based on two arguments. First, the U.S. defense industry has almost no direct import or export relationship with China due to stringent strategic trade regulations. Second, the items subject to this tariff or retaliation actions by China are almost not used in the production process of U.S. defense companies. However, to ensure that my results are not driven by the Section 301 tariffs, I have re-estimated the model, including the key events of this latter tariff scheme as covariates. For this test, I have identified the main key events related to the Section 301 tariffs using the same approach above, yet using the keywords “Section 301” or “Trade Act” in my search query (Table A3 in the Appendix in S1 File lists the key events found). The regression results in Table 4 indicate that none of these Section 301 tariff events have any significant effect. Conversely, the results of the Section 232 tariffs remain almost unaffected. However, it could be argued that the two tariff schemes influence each other. To address any potential contagion effect between the two schemes, I have dropped in column (2) of Table 4 all overlapping events that are within a time window of [+2,-2]. However, it appears that this filter does not affect the main pattern of findings.

**Table 4:** Economic spillovers with Section 301 tariffs.

	(1)		(2)		(3)		(4)	
	Section 232		Section 301		Section 232		Section 301	
Start and progress investigation	-0.0057	**	-0.0015		-0.0054	*	-0.0014	
	(0.001)		(0.001)		(0.008)		(0.002)	
Public release investigation report	-0.0008				-0.0004			
	(0.001)				(0.000)			
Official announcement tariff	-0.0033	**	-0.0007		-0.0010	**	-0.0005	
	(0.001)		(0.001)		(0.001)		(0.000)	
Formal imposition date	-0.0080	*	-0.0020		-0.0028	**	-0.0017	
	(0.004)		(0.002)		(0.001)		(0.002)	
Statement about extending tariff	-0.0040		-0.0012		-0.0011		-0.0014	
	(0.004)		(0.002)		(0.001)		(0.001)	
Announcing retaliation actions	-0.0017	**	-0.0004		-0.0004	**	-0.0003	
	(0.001)		(0.000)		(0.000)		(0.000)	
Start exemption negotiation	0.0034	*			0.0007	*		
	(0.002)				(0.000)			
Exemption granted	0.0022		0.0005		0.0004		0.0006	
	(0.003)		(0.000)		(0.000)		(0.001)	
Exemption withdrawn	-0.0017				-0.0009			
	(0.001)				(0.001)			
Events	ALL				Excluding overlapping events			
Number of observations	61750				61750			

Note: Panel-corrected robust standard errors in parentheses.

*, ** and *** denote statistical significance at the 1%, 5% and 10% level, respectively. All regressions are controlled for heteroskedasticity.

### 5.5 Economic spillovers

The results so far indicate that the introduction of the steel and aluminum tariffs put the economic performance of the U.S. defense industry under a certain downward pressure. However, two questions remain. First, does the reaction of investors in the U.S. defense industry differ from the response of investors in other U.S. sectors? Second, are there international spillovers of these import tariffs for defense companies outside the U.S.?

In an attempt to answer the first question, I estimate my main model, in columns (1) and (2) of Table 5, using data on the U.S. automobile and construction industry, as these industries are also recognized as large-scale steel users (see also [85–87]). However, in contrast to the defense industry, these industries rely to a large extent on imported steel. The results indicate that over my period of analysis, the abnormal stock market returns of the considered automobile and construction companies dropped by about 3.4 and 0.7 percent, respectively. This indicates that the automobile industry is statistically almost in the same range as the accumulated impact on the U.S. defense industry. This finding is quite surprising since the tariff scheme is expected to have a larger negative impact on the automobile industry. This expectation mainly rests on three arguments. First, the adverse impact of expected retaliation measures hampers the economic performance of the automobile industry more due to the intensive international competition, less market power, and the increased availability of alternatives. Second, the price of imported steel becomes relatively less competitive compared to domestically produced steel. Third, the automobile industry is likely to be also affected more by the Section 301 import tariffs from the Trade Act. All these arguments together should put the stock market performance of the automobile industry under more downward pressure than the defense industry. However, one alternative explanation for the finding could be that investors in the U.S. defense industry overestimate the tariff impact on production costs and underestimate the market power of the U.S. defense industry, or are unfamiliar with the primary use of domestically produced in this industry due to incomplete information. In turn, the economic performance of the construction sector is less affected by the tariff scheme than the defense industry. One logical explanation is that the prices of steel and aluminum determine a smaller part of the total costs in this industry.

**Table 5:** Economic spillovers.

	(1)		(2)		(3)		(4)	
Start and progress investigation	-0.0020	**	-0.0006	*	-0.0016		0.0022	
	(0.001)		(0.000)		(0.001)		(0.002)	
Public release investigation report	-0.0004		-0.0001		-0.0002		0.0002	
	(0.001)		(0.000)		(0.000)		(0.000)	
Official announcement tariff	-0.0031	**	-0.0007	*	-0.0009		0.0012	
	(0.001)		(0.000)		(0.001)		(0.001)	
Formal imposition date	-0.0131		-0.0034	*	-0.0038		0.0040	
	(0.021)		(0.002)		(0.003)		(0.004)	
Statement about extending tariff	-0.0034		-0.0010		-0.0010		0.0014	
	(0.006)		(0.002)		(0.002)		(0.003)	
Announcing retaliation actions	-0.0019	**	-0.0005		-0.0004		0.0006	
	(0.001)		(0.001)		(0.000)		(0.001)	
Start exemption negotiation	0.0017		0.0004		0.0009		-0.0010	
	(0.001)		(0.001)		(0.001)		(0.001)	
Exemption granted	0.0005		0.0001		0.0004		-0.0004	
	(0.000)		(0.000)		(0.000)		(0.000)	
Exemption withdrawn	-0.0008		-0.0002		-0.0006		0.0010	
	(0.001)		(0.000)		(0.001)		(0.001)	
Accumulated impact	-3.4%		-0.7%		0.0%		0.0%	
Events	ALL		ALL		ALL		ALL	
Sample of firms	Automobile		Construction		S&P500		European defense firms	
Number of observations	32195		28146		950		21850	

Note: Panel-corrected robust standard errors in parentheses.

*, ** and *** denote statistical significance at the 1%, 5% and 10% level, respectively. All regressions are controlled for heteroskedasticity.

Additionally, in computing the abnormal return, I subtracted the market return from the actual return. However, when the tariff scheme already affects the market return, the results presented so far underestimate the total effect. In that case, the tariff scheme has both a general market effect and a sector-specific effect. To test this notion, I use the daily return of the S&P500 as my dependent variable. Given the relative insignificance of large steel consumers, especially defense firms, in the S&P500 index, the events associated with the steel tariffs are expected to have caused no harm to this index. In column (3) of Table 5, I report the results of this placebo test. My expectations are mainly confirmed since any event category significantly affects the return of the S&P500.

Finally, the defense industrial base significantly differs among countries. The U.S. defense industry is regarded to be highly competitive and dominated by economies of scale, while the defense industry in most other countries is much more fractionalized and thrives on government support [88]. The imposition of the steel tariffs might entail some benefits for defense companies outside of the U.S., for instance, by creating a cost advantage or by filling the gap created in the export market due to some retaliatory measures. To further explore this issue, I explore in column (4) of Table 5 the impact of the tariff events on the abnormal stock market return on a sample of European defense companies. It appears that investors expect these firms not to be significantly affected by the trade policy actions taken by the U.S. administration or the retaliation actions introduced by U.S. trading partners. Thus, investors in the international defense industry do not expect that U.S.-produced military equipment will be replaced by items produced in the rest of the world or that steel prices will substantially be affected. One critical remark regarding this latter analysis is that the samples of U.S. and E.U. firms are not directly comparable. One reason is that the European defense industry is much more dominated by SMEs and privately owned companies. Also, their average firm size (measured by assets, revenue, or number of employees) is substantially lower. Consequently, the E.U. defense industry has considerably less market power in the export market than U.S. companies.

## 6. Conclusion

In 2018, the Trump administration took three types of actions derived from existing U.S. trade laws that are unusual, if not unprecedented. First, it has invoked rarely used rationales for imposing trade barriers by citing national security concerns. Second, the administration has declared its intention to initiate its own investigations and actions rather than wait for companies to request them. Third, it has signaled its willingness to undertake routine, technocratic trade policy responses to low-priced imports while accompanying these steps with overheated political rhetoric that virtually invites retaliation by trade partners.

One sector that was likely to be in particular hurt by these trade policy actions is the defense-related industry, as it is recognized among the largest consumers of steel and aluminum. A question that remains unanswered so far is whether the coercive trade measures on steel and aluminum taken by the Trump administration came as a complete surprise or were already expected and even partly anticipated by investors in the U.S. defense industry. On the one hand, when the tariff imposition came suddenly and unexpectedly, investors were unprepared to cope effectively with the new situation. This would most likely lead to a drop in the stock prices of U.S. defense-related firms almost immediately following the official announcement, as investors expected higher production costs, lower cash flows, and fewer profits in the near future. On the other hand, it is possible that before the formal imposition of the trade tariff, investors were speculating on these protective trade measures.

My analysis reveals that investors perceived the introduction of the steel and aluminum tariffs by President Trump as bad news for the market value of U.S. defense companies. The drop in the cumulative abnormal stock market returns is likely to be caused by a decrease in the expected companies’ future cash flows, higher production costs, and the fear of retaliation actions taken by important trading partners. Even before the Department of Commerce released the findings of its investigation and President Trump formally announced the tariff, investors were speculating on the introduction of steel and aluminum trade restrictions. However, the actual tariff rate introduced exceeded expectations since the negative sentiment was aggravated after the official announcement of the tariff by President Trump. Finally, when comparing the U.S. defense industry with foreign competing companies or other large steel-consuming companies, it appears that the U.S. defense industry plays a unique role due to its dominant position in the export market.

The findings of this study reveal a clear significant trade-off as safeguarding the domestic steel industry using protective trade policies negatively affects the U.S. defense sector. This insight casts doubt on President Trump’s justification for protecting the steel industry to enhance national security. The essential issue is whether these goals can be aligned. Protecting the domestic steel industry carries the risk of causing harm to industries that use steel for production. These steel-using industries are economically far more important as they employ many more people, including the automobile, construction, aerospace, and defense industries.

Nevertheless, the impact of the Trump tariffs was still rather limited for the U.S. defense industry due to their monopoly power. However, in the future this might change as many countries around the world, including the EU, have revised their strategic agenda the last few years and invest much more in their defense industrial base. In the short run, the U.S. defense industry might still reap the benefits from economies of scale and the comparative technologic advantage. However, the pursuit of strategic autonomy by many other countries may, in the long run, weaken the American defense industry. his shift will only be exacerbated by deteriorating trade policies that undermine the competitiveness of the American defense industry. The impact of these rapid changes in the defense industrial base in many countries on the outcome of trade policies, I leave for further future research.

## Supporting information

S1 File Online appendix. (DOCX)
