# Supplementary material for: Trump tariffs and the U.S. defense industry
Source: PLoS One. 2025 Jan 24;20(1):e0313204. doi: 10.1371/journal.pone.0313204 (PMC11759384; doi:10.1371/journal.pone.0313204)
Supplement: S1 File — (DOCX) [file pone.0313204.s001.docx]

**Online appendix**

| Table A1: Key events Section 232 tariffs | | |
| --- | --- | --- |
| Date | Event | Event type |
| 19-4-2017 & 26-4-2017 | President Trump instructs Commerce Secretary Wilbur Ross to self-initiate two investigations into whether steel (April 19) and aluminum (April 26) imports threaten U.S. national security under Section 232 of the Trade Expansion Act of 1962. | Investigation |
| 24-5-2017 | Public hearing in Congress about the investigation by the Department of Commerce. | Investigation |
| 11-1-2018 | Commerce submits steel and aluminum reports to President | Investigation |
| 16-2-2018 | The U.S. Department of Commerce recommends that President Trump boost American production either by imposing a tariff of 24 percent on all steel and aluminum imports, imposing higher tariffs on imports from specific countries, or setting a quota on imports. | Release |
| 1-3-2018 | Trump tweets that ‘trade wars are good’ as he announces the U.S. is preparing to impose metal tariff plans. Trump announces forthcoming tariffs on all trading partners of 25 percent on steel and 10 percent on aluminum under national security grounds. | Release |
| 3-3-2018 | Jean-Claude Juncker, President of the EU’s executive arm, the European Commission (EC), responds by announcing planned retaliatory measures. | Retaliation |
| 8-3-2018 | Trump signs an order for a 25 percent tariff on steel imports and a 10 percent tariff on aluminum to come into effect on May 1 but says exemptions will be negotiated. These exemptions exclude about one-third of the imports announced a week earlier. He decides that partners can negotiate with U.S. Trade Representative to be excluded from the tariffs, and companies can file petitions with Commerce Secretary Ross to have specific products excluded from the tariffs. | Official announcement |
| 18-3-2018 | The U.S. Department of Commerce announced its procedures for excluding products from the recently announced tariffs on steel and aluminum product imports. Only individuals or organizations using steel or aluminum articles identified in Presidential Proclamations 9704 and 9705 and engaged in business activities in the United States may submit exclusion requests. Separate exclusion requests must be submitted for each unique steel or aluminum product import. | Exemption negotiation |
| 23-3-2018 | Trump’s steel and aluminum tariffs go into effect with exemptions for selected countries. There is no timeline or explicit criterion for the removal of the restrictions. Temporary exemptions to May 1 are in place for certain U.S. security partners (later extended to June 1). Trump issues revised formal steel and aluminum tariffs proclamations, further exempting the European Union, South Korea, Brazil, Argentina, and Australia—in addition to Canada and Mexico as previously announced—but only through May 1, 2018. This means another third of the originally covered imports on March 1 are temporarily exempt. | Imposition date |
| 28-3-2018 | Korea agrees to reduce steel exports to the United States in return for a permanent exemption from the steel tariff. | Exemption granted |
| 2-4-2018 | China imposes retaliatory tariffs on aluminum waste and scrap, pork, fruits and nuts, and other U.S. products, worth $2.4 billion in export value in 2017. This compares to the U.S. steel and aluminum tariffs covering Chinese exports worth $2.8 billion in 2017. | Retaliation |
| 9-4-2018 | China initiated a WTO complaint against the U.S. steel and aluminum tariffs. | Retaliation |
| 30-4-2018 | President exempts South Korea permanently from steel duties; However, aluminum imports from South Korea are subject to the additional tariff on that product as of May 1. | Exemption granted Exemption failure |
| 30-4-2018 | The Trump administration extends the steel and aluminum tariffs exemptions provided to the European Union, Canada, and Mexico until June 1, 2018. Korea’s aluminum tariff exemption ends. Argentina, Australia, and Brazil receive indefinite exemptions for steel and aluminum tariffs while finalizing details on “satisfactory alternative means to address the threatened impairment to the national security” by the imports. | Formal announcement tariff |
| 1-5-2018 | The White House says the Trump administration will extend negotiations with the EU and others on exemptions from the steel and aluminum tariffs until June 1. | Exemption negotiation |
| 2-5-2018 | Permanent steel exemption was granted for Argentina, Australia, and Brazil. | Exemption granted |
| 17-5-2018 | President exempts Canada and Mexico from steel and aluminum duties. Canada, Mexico, and United States announced the process for reinstating tariffs should import surge. | Exemption granted |
| 31-5-2018 | President permanently exempts Argentina and Brazil from steel duties, and Argentina from aluminum duties, based on quota arrangements. Australia permanently exempted from both duties without a quota. | Exemption granted |
| 31-5-2018 | President Trump started extending the tariff schedule on imports from Mexico, Canada, and the European Union as of June 1, 2018. | Formal announcement tariff |
| 1-6-2018 | The EU fails to reach an agreement with the U.S. Department of the Treasury on permanent exemptions from steel and aluminum tariffs. The United States moves forward with 25 percent tariffs on steel and 10 percent on aluminum for the European Union, Canada, and Mexico by ending their previously granted exemptions effective June 1. | Exemption failure |
| 5-6-2018 | Mexico's retaliatory tariffs are imposed. | Retaliation |
| 6-6-2018 | The U.S. imposes tariffs on the EU’s steel and aluminum products. The EU says it expects to retaliate in July. | Formal announcement tariff;  Exemption failure |
| 15-6-2018 | Chinese retaliatory tariffs are imposed. | Retaliation |
| 22-6-2018 | EU retaliatory tariffs imposed. The European Union activated its previous tariff threat on the United States, with an initial list covering $3.2 billion of U.S. products in 2017. Steel and aluminum make up 34 percent of the affected products, while the rest are agricultural and food products, and other consumer goods. Specific items include bourbon whiskey, motorboats and yachts, motorcycles, blue jeans, corn, and peanut butter. | Retaliation |
| 1-7-2018 | Canada retaliatory tariffs are imposed. Canada imposed tariffs on U.S. products totaling $12.8 billion in 2017. Half of the targeted goods are steel and aluminum. American agricultural and food products make up 19 percent, and 24 percent are other consumer goods. Steel products face a 25 percent tariff, while the remaining products are hit at a 10 perc ent rate. | Retaliation |
| 1-7-2018 | The EU warns that nearly US$300 billion of U.S. exports could be hit by retaliatory tariffs if the Trump administration decides to penalize car imports from around the world. | Retaliation |
| 6-7-2018 | The second round of Chinese retaliatory tariffs was imposed. | Retaliation |
| 16-7-2018 | The United States launched separate disputes at the World Trade Organization (WTO) against China, the European Union, Canada, Mexico, and Turkey, challenging the tariffs each WTO Member imposed in response to President Trump’s Section 232 actions on trade in aluminum and steel to protect the United States national security interests. Collectively, the five economies have retaliated with tariffs on U.S. exports worth $24 billion in 2017. | Retaliation |
| 11-7-2018 | Multiple countries announce severe retaliatory measures. | Retaliation |
| 16-7-2018 | According to the Office of the U.S. Trade Representative (USTR), the United States launched separate disputes at the World Trade Organization (WTO) against China, the European Union, Canada, Mexico, and Turkey, challenging the tariffs each WTO Member imposed in response to President Trump’s Section 232actions on trade in aluminum and steel to protect the United States national security interests. | Retaliation |
| 25-7-2018 | At a White House meeting between Trump and Juncker, the U.S. lifted the metal tariffs on Europe and promised to hold off on further tariffs. In return, Juncker says Europe will import more American soybeans and facilitate imports of liquefied natural gas. The pair also vow that the U.S. and EU will work together toward ‘zero tariffs, zero non-tariff barriers, and zero subsidies on non-auto industrial goods. | Exemption granted |
| 8-4-2018 | Indian retaliatory tariffs were imposed. | Retaliation |
| 9-4-2019 | The U.S. announces plans to target US$25 billion of European exports with tariffs, as part of a dispute over what it says are unfair EU subsidies to large European civil aircraft manufacturers, such as Airbus. Targeted items include cheeses and wine, as well as helicopters and planes. | Retaliation |
| 15-4-2019 | EU ambassadors finally authorized the European Commission to start trade talks with the U.S., following the Juncker–Trump agreement in July 2018. The scope of agreed negotiating directives includes seeking to eliminate duties for industrial goods, excluding agriculture. | Retaliation |
| 17-4-2019 | The European Commission says it wants to impose tariffs on about €20 billion of U.S. exports in retaliation for what it says are ‘unlawful’ subsidies given by the U.S. to American aircraft manufacturer Boeing. | Retaliation |
| 17-5-2019 | President exempts Canada and Mexico from steel and aluminum duties. Canada, Mexico, and United States announced the process for reinstating tariffs should import surge. Canada will remove tariffs levied on American goods in retaliation for the steel and aluminum duties. The United States agreed to remove steel and aluminum tariffs on Canada and Mexico. | Exemption granted |
| 15-6-2019 | India implements retaliatory tariffs against U.S. exports in response to Trump’s steel and aluminum tariffs of March 2018. India had announced the tariffs in mid-2018. Reports tie India’s action to the Trump administration’s decision on June 5, 2019, to increase tariffs on India by removing the country from the U.S. Generalized System of Preferences program for developing countries. | Retaliation |
| 14-10-2019 | President announces plans to increase the steel tariffs on imports from Turkey to 50 percent in response to Turkish military actions but later suspends the planned increase. | Formal announcement tariff |
| 2-12-2019 | President threatens to reinstate steel and aluminum tariffs on imports from Argentina and Brazil due to currency issues. | Formal announcement tariff |
| 24-1-2020 | Trump imposes new tariffs on almost $450 million of steel and aluminum products to help industries suffering from his previous tariffs. They mostly hit imports from allies such as Taiwan, Japan, and the European Union, as well as China. President proclaims certain derivative products of steel and aluminum (e.g., nails, wires, and select motor vehicle body parts) subject to 25% and 10% tariffs, respectively, effective February 8, 2020. | Formal announcement tariff |
| 6-8-2020 | Trump announced the return of 10 percent tariffs on primary aluminum products from Canada, going into effect on August 16. The Canadian government plans to retaliate on a proportionate amount of U.S. exports to Canada of products containing aluminum. | exemption failure; Retaliation |
| 15-9-2020 | The U.S. resumes tariff-free treatment of non-alloyed, unwrought aluminum from Canada retroactive to September 1, 2020, provided Canada restricts exports of those products. Canada withdraws its planned retaliation but denies it agreed to export quotas. | exemption granted |

| Table A2: U.S. Defense companies included | | |
| --- | --- | --- |
| Advanced Tchn. Prds. | Esco Technologies | Olin |
| Aerojet Rocketdyne | General Dynamics | Orbital Sciences |
| Allegheny Technolo | General Electric | Oshkosh Truck |
| Alliant Technologies systems | Grumman | Raytheon |
| Allied Defense Group | Halliburton | Ride |
| Anteon corporation | Harris | Robot Defense Systems |
| Armor Hdg. | Heico Corp HEI | Rockwell Collins |
| Ball | Hi Shear Technology | Sequa |
| Boeing | Honeywell | Silicon Graphics |
| BWX Technologies | Huntington Ingalls Industries | Stewart & Stevenson |
| CACI | Integrated Defense Technologies | Sturm Ruger & Co |
| Colt Industries | Itt Industries | Tactical Air Defense Services |
| Computer Scis. | Jacobs Engineering Group | Teledyne Technologies |
| Cordant Technologies | Kratos Defense & Security solutions | Textron Inc |
| Cubic | L-3 Communications | Titan |
| Curtiss Wright | L3Harris Technolog | TransDigm Group |
| Defense Technology Systems | Lockheed Martin | Triumph Group Inc |
| Drs Technologies | Mantech International | United Technologies |
| Ducommun | Mcdonnell Douglas | URS |
| Ducommun Incorpora | Mercury Systems Inc. | Washington Group International |
| Dynamic Materials | Military Communications Technologies |  |
| Dyncorp | Moog |  |
| EDO | Northrop Grumman |  |

| Table A3: Key events Section 301 tariffs | | |
| --- | --- | --- |
| Date | Event | Event type |
| 14-8-2017 | U.S. President Donald Trump orders “Section 301” probe into alleged Chinese intellectual property theft. | Investigation |
| 22-1-2018 | President Trump imposes tariffs on all imported washing machines and solar panels. These tariffs apply to all imports, not just those from China. | Official announcement |
| 18-4-2018 | China responded to US Section 301 List 1 tariffs with a two-part list of countermeasures. | Retaliation |
| 16-6-2018 | China responded to additional US Section 301 List 2 tariffs with an additional two-part list of countermeasures | Retaliation |
| 20-6-2018 | U.S. imposes List 1 tariffs with a 25% levy on $34 billion of Chinese imports. U.S. proposes List 2 tariffs with a 25% levy on $16 billion of Chinese imports. | Official announcement |
| 6-7-2018 | Expansion of retaliation measures by China to List 1 | Retaliation |
| 10-7-2018 | U.S. proposes List 3 tariffs with a 10% levy on $200 billion of Chinese imports. | Official announcement |
| 1-8-2018 | President Trump announces a List 3 tariff increase from 10 to 25%. | Official announcement |
| 16-8-2018 | U.S. officially releases List 2. China retaliates with 25% duties on $16 billion of U.S. imports. | Official announcement |
| 23-8-2018 | Extension of countermeasures by China to List 2 | Retaliation |
| 24-9-2018 | U.S. indicates the List 3 tariff rate will increase to 25% on January 1, 2019. China retaliates with duties on $60 billion of U.S. imports. | Official announcement |
| 1-12-2018 | U.S. and China agree to a 90-day halt on new tariffs. U.S. agrees to delay scheduled List 3 tariff increase until March. China agrees to buy a “very substantial” amount of U.S. products. | Exemption granted |
| 13-5-2019 | U.S. proposes List 4 tariffs with 25% levy on $300 billion of Chinese imports. China retaliates by increasing duties on $60 billion worth of U.S. imports. | Official announcement |
| 1-6-2019 | Revision of Chinese countermeasures against List 3. | Retaliation |
| 14-8-2019 | USTR splits List 4 into List 4A and List 4B. | Official announcement |
| 23-8-2019 | China announced it will impose tariffs ranging from 5 to 10% on $75 billion worth of U.S. goods. | Retaliation |
| 26-8-2019 | President Trump retaliates announcing a 5% tariff increase on all Chinese imports and ordering all American companies to cease manufacturing in China | Official announcement |
| 11-10-2019 | U.S. postpones 5% tariff increase on $250 billion of Chinese goods citing advancement in trade talks between the two nations. China agrees to purchase $50 billion worth of U.S. agricultural products. | Exemption granted |

| Table A4: Descriptive statistics | | | |
| --- | --- | --- | --- |
|  | Obs | Mean | St. dev |
| Abnormal return | 65296 | 0.00071 | 0.016 |
| Volatility index (VIX) | 65296 | 18.23 | 9.72 |
| Change in the exchange rate index | 65296 | -0.001 | 0.012 |
| Covid-19 dummy | 65296 | 0.21 | 0.41 |
| Jamal Khashoggi dummy | 65296 | 0.57 | 0.49 |
| Asset size (logarithm) | 65296 | 19.24 | 6.23 |
| Daily volume traded (logarithm) | 65296 | 10.84 | 3.84 |
| Start and progress investigation | 65296 | 0.006 | 0.075 |
| Public release investigation report | 65296 | 0.004 | 0.062 |
| Official announcement tariff | 65296 | 0.002 | 0.043 |
| Formal imposition date | 65296 | 0.002 | 0.043 |
| Statement about extending tariff | 65296 | 0.013 | 0.115 |
| Announcing retaliation actions | 65296 | 0.031 | 0.172 |
| Start exemption negotiation | 65296 | 0.006 | 0.075 |
| Exemption granted | 65296 | 0.013 | 0.115 |
| Exemption withdrawn | 65296 | 0.006 | 0.0075 |
